# All Kinds of Sunny Colors Synthesized from Methane: Genome-Encoded Carotenoid Production by *Methylomonas* Species

**DOI:** 10.3390/microorganisms11122865

**Published:** 2023-11-26

**Authors:** Igor Y. Oshkin, Ekaterina N. Tikhonova, Ruslan Z. Suleimanov, Aleksandr A. Ashikhmin, Anastasia A. Ivanova, Nikolai V. Pimenov, Svetlana N. Dedysh

**Affiliations:** 1 Winogradsky Institute of Microbiology, Research Center of Biotechnology of the Russian Academy of Sciences, Moscow 117312, Russia; 2 Institute of Basic Biological Problems, Pushchino Scientific Center for Biological Research of the Russian Academy of Sciences, Pushchino 142290, Russia

**Keywords:** carotenoids, lycopene, methanotrophic bacteria, pigmented *Methylomonas*, carotenoid-containing feed protein, genome analysis

## Abstract

Carotenoids are secondary metabolites that exhibit antioxidant properties and are characterized by a striking range of colorations from red to yellow. These natural pigments are synthesized by a wide range of eukaryotic and prokaryotic organisms. Among the latter, carotenoid-producing methanotrophic bacteria, which display fast growth on methane or natural gas, are of particular interest as potential producers of a feed protein enriched with carotenoids. Until recently, *Methylomonas* strain 16a and *Methylomonas* sp. ZR1 remained the only representatives of the genus for which detailed carotenoid profile was determined. In this study, we analyzed the genome sequences of five strains of *Methylomonas* species whose pigmentation varied from white and yellow to orange and red, and identified carotenoids produced by these bacteria. Carotenoids synthesized using four pigmented strains included C30 fraction, primarily composed of 4,4’-diaplycopene-4,4’-dioic acid and 4,4’-diaplycopenoic acid, as well as C40 fraction with the major compound represented by 1,1’-dihydroxy-3,4-didehydrolycopene. The genomes of studied *Methylomonas* strains varied in size between 4.59 and 5.45 Mb and contained 4201–4735 protein-coding genes. These genomes and 35 reference *Methylomonas* genomes available in the GenBank were examined for the presence of genes encoding carotenoid biosynthesis. Genomes of all pigmented *Methylomonas* strains harbored genes necessary for the synthesis of 4,4’-diaplycopene-4,4’-dioic acid. Non-pigmented “*Methylomonas montana*” MW1^T^ lacked the *crtN* gene required for carotenoid production. Nearly all strains possessed phytoene desaturases, which explained their ability to naturally synthesize lycopene. Thus, members of the genus *Methylomonas* can potentially be considered as producers of C30 and C40 carotenoids from methane.

## 1. Introduction

Carotenoids are a class of abundant naturally occurring pigments synthesized by all photosynthetic organisms and some non-photosynthetic bacteria and fungi [1]. To date, the Carotenoids Database (http://carotenoiddb.jp, accessed on 20 November 2023) [2] provides information on the chemical structures of more than 1200 carotenoids found in >720 organisms from all domains of life. This diversity and ubiquity establish carotenoids as the second most prevalent natural compounds after chlorophyll [3]. Carotenoids are isoprenoid compounds with a polyene backbone that contains a variable number of conjugated double bounds (with a maximum absorbance of 400–500 nm), a feature that confers coloration in the yellow-to-red range [4].

Carotenoids can be categorized into two primary groups based on the oxygenation status: (i) oxygenated molecules or xanthophylls (e.g., astaxanthin, canthaxanthin, lutein, zeaxanthin) and (ii) non-oxygenated molecules or carotenes (e.g., lycopene, α-carotene, β-carotene) [5]. Carotenoids are highly sensitive to oxygen, heat, and light, as well as acidic or basic conditions [6,7]. The precursors for carotenoid biosynthesis, isopentenyl pyrophosphate (IPP), and its isomer dimethylallyl pyrophosphate (DMAPP) are generated through two distinct metabolic routes, i.e., the mevalonate pathway and non-mevalonate (MEP/DOXP) pathways [8,9]. Isoprenoid synthesis in prokaryotes predominantly relies on the MEP/DOXP pathway. The mevalonate pathway is rarely present and only a few prokaryotes have both [10]. Chain elongation from C10 to C20 occurs through the successive head-to-tail condensation of IPP to DMAPP, resulting in the synthesis of geranyl pyrophosphate (GPP), farnesyl pyrophosphate (FPP), and geranylgeranyl diphosphate (GGPP) [11]. Consequently, carotenoids originating from C30 and C40 chains follow distinct metabolic routes. C30 carotenoid synthesis proceeds with the addition of two FPP units, whereas C40 carotenoid synthesis is driven by the incorporation of two GGPP units. Carotenoids belonging to the C30 group are subject to early functionalization through oxidation processes, resulting in the production of aldehyde, ketone, and/or carboxyl groups. In contrast, carotenoids in the C40 group undergo dehydrogenation and cyclization processes prior to these oxidation reactions [7].

Currently, 179 representatives of the domain *Bacteria* have been described as a source of carotenoids. However, the number of pigmented bacteria is much bigger. Among them are aerobic methanotrophs, characterized by the unique ability to utilize methane (CH_4_) as their sole source of energy. Methanotrophs possess methane monooxygenase, the enzyme that can activate the stable C-H bond in the CH_4_ molecule (104 kcal/mol), which explains its unique ability to use methane for growth [12,13,14]. The currently described diversity of aerobic methanotrophs encompasses ~30 genera within *Gamma*- and *Alphaproteobacteria* and the *Verrucomicrobia* [15], many of which are represented by pigmented phenotypes. In particular, the presence of pigments is one of the genus-specific features of *Methylomonas* species [16]. The only exception is “*M. montana*”, which was described as a non-pigmented methanotroph [17]. Growing interest in the field of methanotrophic biocatalysis is driven by the availability and abundance of methane as a component of natural gas. Methanotrophs have been intensively exploited for the conversion of CH_4_ to single-cell protein [18,19] and various value-added products, including methanol [20], polyhydroxybutyrate [21], lipids [22], and organic acids [23]. High biotechnological potential has been shown for *Methylomonas* and *Methylococcus* genera, which accommodate fast-growing methanotrophs [24,25,26]. Thus, *Methylococcus capsulatus* was used for a large-scale commercial production of microbial proteins from natural gas [19,27]. *Methylomonas* species were also considered as potential producers of a single-cell protein, i.e., protein produced in microbial cells [28,29]. Notably, *Methylomonas* bacteria have a clear advantage over non-pigmented *Methylococcus* methanotrophs due to the presence of carotenoids in their biomass. Recently described *Methylomonas rapida* MP1^T^ displayed a high growth rate in a bioreactor supplied with natural gas and produced both C30 (4,4’-diaplycopene-4,4’-dioic acid, 4,4’-diaplycopenoic acid) and C40 (1,1’-dihydroxy-3,4-didehydrolycopene) carotenoids [30]. Few studies described the construction of the metabolic route leading to C40 carotenoid, astaxanthin, in *Methylomonas* hosts [31,32]. This was implemented by blocking the production of C30 carotenoids via gene deletions and via the subsequent introduction of the C40 carotenoid biosynthesis genes (*crtE, crtY, crtI, crtB, crtW,* and *crtZ*).

The biosynthesis of C30 carotenoids in methanotrophic bacteria was studied on the basis of *Methylomonas* sp. 16a. Tao et al. [33] identified the *crtNb* gene involved in the conversion of 4,4’-diapolycopene to 4,4’-diapolycopene aldehyde, as well as the aldehyde dehydrogenase gene (*ald*) responsible for the subsequent oxidation of 4,4’-diapolycopene aldehyde to 4,4’-diapolycopene acid. The group of C30 4,4’-diapocarotenoids also encompasses compounds like 4,4’-diapocarotene-4-oic acid and fatty acid esters di-(β,D-glucosyl)-4,4’-diapocarotene-4,4’-dioate from *Methylobacterium rhodinum* [34]. It also includes compounds like 4,4’-diaponeurosporene and OH-diaponeurosporene found in *Heliobacteria* [35,36], as well as staphyloxanthin, 4,4’-diapophytoene, 4,4’-diapophytofluene, 4-4’-diapo-zeta-carotene, 4,4’-diaponeurosporene, and its derivatives from *Staphylococcus aureus* [37,38].

In this study, we obtained three novel isolates of *Methylomonas* bacteria and sequenced their genomes. The pool of studied bacteria was expanded by the recently described red-colored *M. rapida* [30] and non-pigmented “*M. montana*” [17]. The objective of this study was to identify carotenoid composition in these *Methylomonas* strains and to examine genomes of these bacteria for the presence of genes related to carotenoid biosynthesis.

## 2. Materials and Methods

### 2.1. Strains and Cultivation Procedures

Previously described representatives of the genus *Methylomonas*, *M. rapida* MP1^T^ [30], and “*M. montana*” MW1^T^ [17], as well as three newly isolated strains of *Methylomonas* species, MY1, MO1, and MV1, were used in this study. New isolates were obtained from surface (0–2 cm) sediment samples collected in 2021 and 2022 from various freshwater bodies. Aliquots of sediment samples were used as inoculum to obtain enrichment cultures of methanotrophic bacteria. The latter were obtained using dilute NMS medium (dNMS), containing (in grams per liter of distilled water) KNO_3_, 0.1; MgSO_4_ × 7H_2_O, 0.2; CaCl_2_ × 2H_2_O, 0.04; 100 mM phosphate buffer, pH 5.8, 1% (*v/v*); and a trace element solution 0.1% (*v*/*v*) containing the following (g/L): EDTA, 5; FeSO_4_ × 7H_2_O, 2; ZnSO_4_ × 7H_2_O, 0.1; MnCl_2_ × 4H_2_O, 0.03; CoCl_2_ × 6H_2_O, 0.2; CuSO_4_ × 5H_2_O, 0.1; NiCl_2_ × 6H_2_O, 0.02; and Na_2_MoO_4_, 0.03. One gram of sediment was added to a 500 mL bottle containing 100 mL of dNMS medium. The bottle was sealed with silicone rubber septa, and methane was added aseptically using a syringe equipped with a disposable filter (0.22 µm) to achieve a 10–20% mixing ratio in the headspace. The incubation was performed on a rotary shaker (120 r.p.m.) at 30–35 °C. After 10 days of incubation, the cultures enriched with methanotrophic bacteria were subjected to serial dilutions. After several serial dilution steps, cell suspensions were plated on agar-solidified dNMS medium. The plates were incubated at 30 °C in desiccators containing approximately 30% (*v*/*v*) methane in air. The colonies appearing on the plates were picked and re-streaked on the same agar medium. The set of finally selected colonies was subjected to several additional serial dilution steps in a liquid dNMS medium at 30–35 °C until isolates of methanotrophic bacteria were obtained. Culture purity was verified via examination using phase-contrast microscopy and by plating on 10-fold-diluted Luria–Bertani agar (1.0% tryptone, 0.5% yeast extract, 1.0% NaCl).

### 2.2. Morphological Characterization and Growth Tests

Morphological observations and cell-size measurements were made with a Zeiss Axioplan 2 microscope and Axiovision 4.2 software (Zeiss, Oberkochen, Germany). Electron microscopy was conducted as described previously [30]. Briefly, exponentially grown cells were collected, pre-fixed with 2.5% glutaraldehyde in 0.05 M cacodylate buffer (pH 7.2), and fixed in OsO_4_ (1% OsO_4_ + 0.7% ruthenium red solution) in the same buffer. After fixation, samples were sequentially treated with a 3% uranyl acetate solution and 70% ethanol. Dehydration was performed in 96% ethanol and absolute acetone before embedding in Epon 812 epoxy resin. Ultrathin sections were cut, stained with 3% uranyl acetate and lead citrate, and examined using a JEM 100CXII transmission electron microscope (Jeol, Tokyo, Japan) at an 80 kV accelerating voltage. A comparative analysis of growth characteristics was carried out by observing the growth dynamics of the studied strains in a liquid mineral medium containing 20% methane in the headspace within the temperature range of 4–48 °C. All incubations were performed in triplicate.

### 2.3. Identification of Carotenoids

Centrifuged cell pellets (40–120 mg fresh weight) were extracted with ethanol. The extract was dried on a rotary evaporator. Then, the pigment film was dissolved in 50 µL of a mixture of acetone and methanol (7:2) and 20 µL of this mixture was used to identify carotenoids via HPLC with diode array detection. The HPLC device (Shimadzu, Kyoto, Japan) consisted of (1) a pump LC-10ADVP (Shimadzu, Kyoto, Japan) with a module FCV-10ALVP, (2) a detector with a diode matrix SPD-M20A, and (3) a thermostat CTO-20 AC. The separation of the carotenoids was performed on a 4.6 × 250 mm reversed phase column (Agilent Zorbax SB-C18, Agilent, Santa Clara, CA, USA) at 22 °C. The solvent gradient was described elsewhere [39]. The feed rate of all solvents was 1.0 mL/min. The obtained data were processed using the Origin8 program (OriginLab Corporation, Northampton, MA, USA). The carotenoids were identified by their retention time and absorption spectra [33,34,40,41,42]. The quantification of each carotenoid was performed by comparing its peak area in the region of 360–800 nm to the sum of all carotenoid peaks taken as 100% and was calculated with the LC-solution program (Shimadzu, Kyoto, Japan) using molar extinction coefficients [43].

### 2.4. DNA Extraction

Cultures of new isolates were grown in the liquid dNMS as described above. The cells were harvested after incubation at 30–35 °C on a rotary shaker at 150 rpm for 2 days. In total, 30 mL of cell suspensions were centrifuged at 10,000× *g*. Genomic DNA extraction was completed using the standard CTAB and phenol-chloroform protocol [44]. Briefly, the pellet was resuspended in 567 µL TE buffer. In total, 30 µL of 10% SDS and 3 µL of 20 mg/mL proteinase K were added, and the mixture was incubated at 37 °C for 1 h. Then, 100 µL of 5 M NaCl and 80 µL of CTAB/NaCl solution were sequentially added, with mixing after each addition. The resulting mixture was incubated at 65 °C for 10 min. An equal volume (0.7 to 0.8 mL) of chloroform/isoamyl alcohol was added, mixed, and centrifuged for 5 min at 10,000× *g*. The supernatant was transferred to a new tube and an equal volume of phenol/chloroform/isoamyl alcohol was added. After a 5 min centrifugation, the pellet was resuspended in 0.6 volume of isopropanol, centrifuged at 10,000× *g* for 5 min, and washed with 70% ethanol. After centrifugation, ethanol was discarded, and the pellet was redissolved in nuclease-free water.

### 2.5. Genome Sequencing and Annotation

The genomic paired-end (2 × 300) library was prepared with a NEBNext ultra II DNA Library kit (New England Biolabs, Ipswich, MA, USA) and sequenced using a MiSeq instrument (Illumina, San Diego, CA, USA). Adapter removal and quality filtering was completed using Trimmomatic [45]. Nanopore sequencing library was prepared using the 1D ligation sequencing kit (SQK-LSK108, Oxford Nanopore Technologies, Oxford, UK). Sequencing was performed on an R9.4 flow cell (FLO-MIN106, Oxford Nanopore Technologies, Oxford, UK) using a MinION device (Oxford Nanopore Technologies, Oxford, UK). Hybrid assembly of short and long reads was performed using Unicycler version 0.4.8 [46]. Assemblies were evaluated with Quast 5.0 [47] and Busco 5.1.2 [48]. Annotation was performed using PROKKA version 1.14.5 [49] and BlastKOALA version 3.0 [50].

### 2.6. Phylogenomic Analysis

The genome-based tree of the five *Methylomonas* strains and phylogenetically related members of the family *Methylococcaceae* was reconstructed using the Genome Taxonomy Database and GTDB-Tk version 2.3.2 (https://github.com/Ecogenomics/GtdbTk, accessed on 1 November 2023). The maximum likelihood tree was constructed using MEGA software version 10.2 [51]. The Pyani program version 0.2.12 was used to estimate average nucleotide identities (ANIs) across *Methylomonas* genomes [52].

### 2.7. Identification of Carotenoid Biosynthesis Genes

Reference amino-acid gene sequences of C30 and C40 carotenoid biosynthesis enzymes were retrieved from the “UniProtKB reference proteomes plus Swiss-prot” database [53] and from the KEGG database [54]. The analysis was primarily focused on gene categories related to the biosynthesis of C5, C15, and C20 building blocks as well as the genes involved in the biosynthesis of C30 and C40 carotenoids. The identification of carotenoid biosynthesis genes was conducted using BLASTP version 2.15.0 [55] against a local database that included genomes of five *Methylomonas* strains from this study and RefSeq *Methylomonas* genomes retrieved from GenBank [56]. A gene was considered homologous if a blast hit with E-value < 10^−5^ produced an alignment with a minimum of 50% sequence identity and 50% sequence similarity [57]. Conservation of function was verified by searching against Pfam database [58] and running HMMSCAN [59]. Binary data of presence/absence matrix were plotted using custom script in Python version 3.12.0 (http://www.python.org, accessed on 10 October 2023).

### 2.8. Sequence Accession Numbers

The 16S rRNA gene sequences of strains MO1, MY1, and MV1 have been deposited in the GenBank under the accession numbers OR234854-OR234856, respectively. The assembled genome sequences of strains MO1, MY1, and MV1 have been deposited in NCBI GenBank under the accession numbers JAVSMZ000000000.1, CP133985.1, and JAVSMY000000000.1, respectively.

## 3. Results

### 3.1. Identification and Characterization of Novel Isolates

The spectrum of examined methanotrophs included *M. rapida* MP1^T^, “*M. montana*” MW1^T^, and three new isolates obtained and identified in this study. Strain MO1 was isolated from a sediment of Meshchersky pond in the Moscow region (Russia) and was closely related to “*M. denitrificans”* FJG1 (99.67% 16S rRNA gene sequence similarity) and *M. methanica* MC09 (97.32% 16S rRNA gene sequence similarity). Strain MV1 was obtained from a sediment of an unnamed freshwater pond in the Krasnodar region, South Russia, and displayed 97.91% 16S rRNA gene sequence similarity to *M. koyamae* Fw12E-Y. Finally, strain MY1 was isolated from a sediment of an unnamed freshwater lake in the Krasnodar region and displayed 100% 16S rRNA gene sequence similarity to *M. koyamae* Fw12E-Y. Cells of strains MV1 and MO1 were represented by short rods, 0.7–0.9 μm in length, while cells of strain MY1 were up to 1.5 μm in length (Table 1).

**Figure 1:**
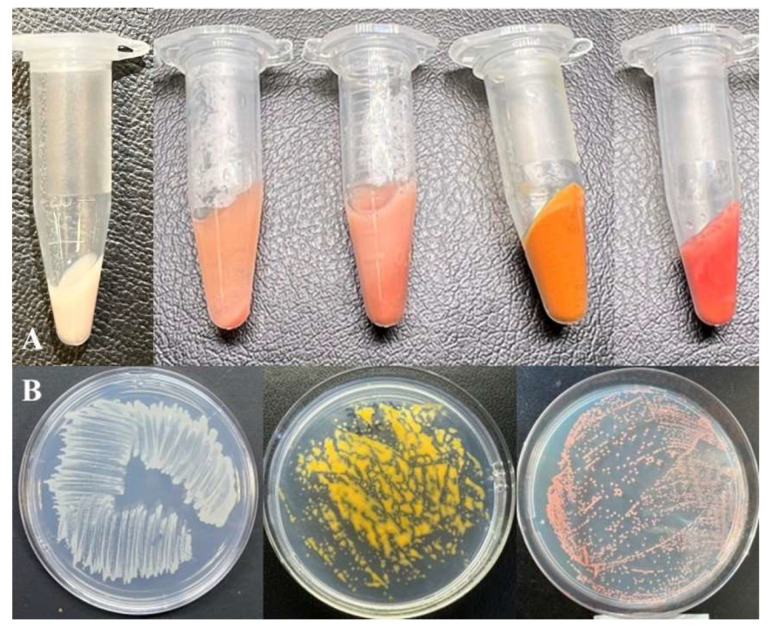
Color spectrum of new *Methylomonas* strains. (**A**) Centrifuged biomass of strains, from left to right: MW1^T^, MV1, MO, MY1, and MP1^T^. Growth of strains on plates with agar medium, from left to right: strains MW1^T^, MY1, and MV1 (**B**).

The slimy colonies formed by these isolates on agar dNMS medium after seven days of incubation with 20% (*v*/*v*) methane were 2–3 mm in diameter, round, and colored as orange (for strain MO1), yellow (for strain MY1), or pink to red (for strain MV1) (Figure 1).

Three novel strains and “*M. montana*” MW1^T^ were capable of growth in a broad temperature range from below 10 °C to 37–38 °C, with an optimum temperature at 30–32 °C. *M. rapida* MP1^T^ was capable of growth up to 45 °C, with an optimum temperature at 35 °C. Specific growth rates of new *Methylomonas* isolates ranged from 0.25 to 0.29 h^−1^, with the highest value (0.29 h^−1^) observed for *M. rapida*. Among all the studied *Methylomonas* representatives, the lowest growth rate (0.13 h^−1^) was observed for “*M. montana*” MW1^T^, while the highest growth rate (0.29 h^−1^) was recorded for *M. rapida* MP1^T^ (Table 1).

### 3.2. Carotenoid Profiles

Carotenoids were detected in the pigment extracts of all strains except for strain MW1^T^. C30 carotenoids were predominant in the pigment fraction, with lesser amounts of C40 carotenoids also identified. The major carotenoid detected in colored isolates was 4,4’-diaplycopene-4,4’-dioic acid (C30). Strains MY1, MO1, and MP1 also contained 1,1’-dihydroxy-3,4-didehydrolycopene (C40) as the second major carotenoid (Table 2).

**Figure 2:**
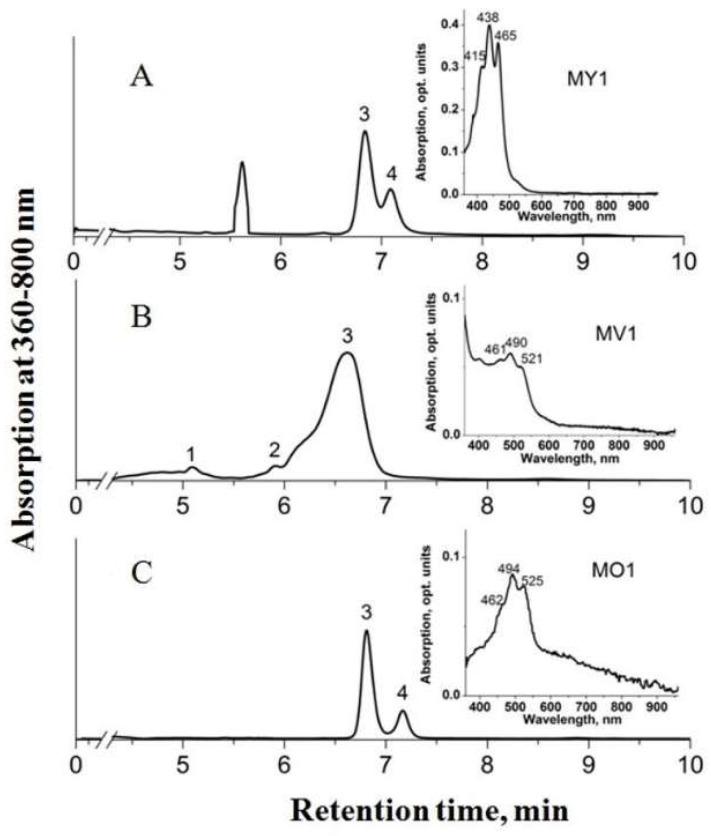
HPLC chromatogram of pigment extracts from strains MY1 (**A**), MV1 (**B**), and MO1 (**C**). Peak identification: 1—1,2-dihydro-3,4-dehydrolycopene; 2—4’-apo-3,4-didehydrolycopene; 3—4,4’-diaplycopene-4,4’-dioic acid; 4—1,1’-dihydroxy-3,4-didehydrolycopene. Inserts: absorption spectra of pigment extracts dissolved in ethanol.

Minor amounts of 4,4’-diaplycopenoic acid (C30), 4’-apo-3,4-didehydrolycopene (C35), and tetradehydrolycopene (C40) were identified in strain MP1^T^. Strain MV1 lacked 1,1’-dihydroxy-3,4-didehydrolycopene, but contained minor amounts of another C40 carotenoid, 1,2-dihydro-3,4-dehydrolycopene, and the C35 carotenoid 4’-apo-3,4-didehydrolycopene. Thus, C35 carotenoids were exclusively present in pigment fraction of strains MV1 and MP1^T^. The HPLC chromatogram of pigment extracts from strains MY1, MV1, and MO1 is shown in Figure 2.

### 3.3. Genome Sequencing and Assembly

The genomes of three novel isolates were sequenced using a hybrid approach. Oxford Nanopore sequencing yielded 211,318–258,525 reads with a total length of 1.2–1.4 Gb (Table 3).

Sequencing on Illumina MiSeq platform generated a total of 211,318–258,525 paired-end reads (300 bp) with a total length of 0.6–1.1 Gb. Both short and long reads were combined to perform a hybrid assembly. The genome of strain MY1 was assembled into a circular contig. Genome assembly for strains MO1 and MV1 consisted of 2 (N50 = 4.93 Mbp) and 10 (N50 = 2.78 Mbp) contigs. The genome characteristics of studied strains are summarized in Table 4.

Genome sizes varied from 4.95 Mb in strain MY1 to 5.42 Mb in strain MV1. The DNA G + C content ranged from 51.44% to 56.16%. *M. rapida* MP1^T^ possessed four copies of rRNA operon, while other *Methylomonas* genomes contained only three copies of rRNA operon. Each genome contained one copy of the pMMO-encoding gene cluster. The number of coding sequences varied between 4201 and 4735.

### 3.4. Genome-Based Phylogeny and Genome-to-Genome Comparison

**Figure 3:**
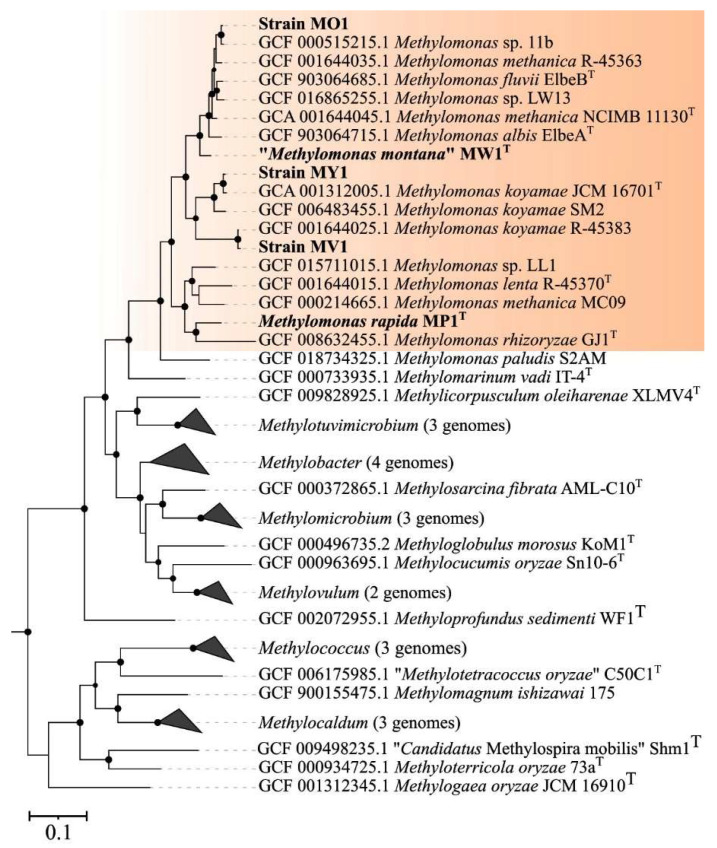
Genome-based phylogeny showing the position of new isolates in relation to other gammaproteobacterial methanotrophs based on the comparative sequence analysis of 120 ubiquitous single-copy proteins. The clade of *Methylomonas* methanotrophs is highlighted in orange. The tree was constructed using the GTDB-Tk version 2.3.2 (https://github.com/Ecogenomics/GtdbTk, accessed on 1 November 2023). The significance levels of interior branch points obtained in maximum-likelihood analysis were determined by bootstrap analysis (100 data re-samplings). Bootstrap values of >70% are shown. The root (not shown) is composed of 52 genomes affiliated to type II methanotrophs *Methylocella*, *Methylocapsa*, *Methyloferula*, *Methylocystis*, and *Methylosinus*). Bar, 0.1 substitutions per amino acid position.

The genome-based phylogeny of new *Methylomonas*-affiliated isolates was elucidated using the comparative sequence analysis of 120 ubiquitous single-copy proteins (Figure 3).

Strains MV1 and MY1 belonged to the phylogenetic clade defined by members of the species *M. koyamae*. Strain MO1 was positioned close to an uncharacterized *Methylomonas* species within a well-supported clade that also included *M. fluvii* ElbeB, *M. methanica* NCIMB 11130, and *M. albis* ElbeA^T^. “*M. montana*” MW1^T^ constituted a separate branch located near the aforementioned clade. The average nucleotide identity (ANI) values were estimated for each pair within a set comprising publicly available *Methylomonas* genomes and genomes of studied strains. Below are the data on the pairwise comparison of ANI values between the new isolates and closely related taxonomically characterized methanotrophs. ANI values calculated for strains MY1 and *M. koyamae* JCM 16701^T^ and for strain MV1 in comparison to *M. koyamae* JCM 16701^T^ constituted 96.8% and 77.9%, respectively. The determined ANI value for strain MO1 and *M. fluvii* EbB was 89.0%. The fact that ANI values between the genomes of strains MV1, MO1, and closely related methanotrophs were estimated below a 95% threshold indicates that these novel isolates may potentially represent new species within the genus *Methylomonas*, according to current standards for using genome data in the taxonomy of prokaryotes [60,61,62].

### 3.5. Analysis of Carotenoid Biosynthesis Genes

**Figure 4:**
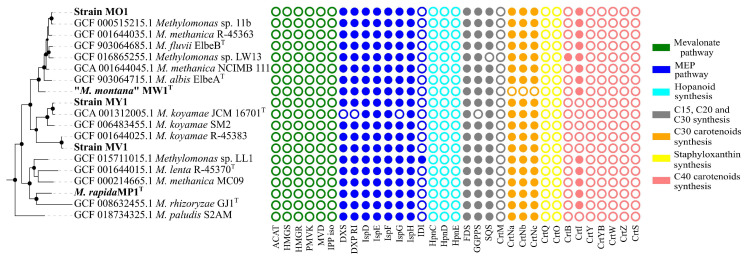
Distribution of genes related to carotenoid biosynthesis in *Methylomonas* genomes. Abbreviations for genes: ACAT, acetyl-CoA C-acetyltransferase; HMGS, hydroxymethylglutaryl-CoA synthase; HMGR, hydroxymethylglutaryl-CoA reductase (NADPH); PMVK, phosphomevalonate kinase; MVD, diphosphomevalonate decarboxylase; IPP iso, isopentenyl-diphosphate delta-isomerase; DXS, 1-deoxy-D-xylulose-5-phosphate synthase; DXS RI, 1-deoxy-D-xylulose 5-phosphate reductoisomeras; ispD, 2-C-methyl-D-erythritol 4-phosphate cytidylyltransferase; ispE, 4-diphosphocytidyl-2-C-methyl-D-erythritol kinase; ispF, 2-C-methyl-D-erythritol 2,4-cyclodiphosphate synthase; IspG, (E)-4-hydroxy-3-methylbut-2-enyl-diphosphate synthase; IspH, 4-hydroxy-3-methylbut-2-en-1-yl diphosphate reductase; IDI, isopentenyl-diphosphate Delta-isomerase; HpnC, hydroxysqualene_synthase; HpnD, presqualene_diphosphate_synthase; HpnE, hydroxysqualene_dehydroxylase; FDS, farnesyl diphosphate synthase; GGPPS, geranylgeranyl diphosphate synthase; SQS, squalene synthase; CrtM, diapophytoene synthase; CrtNa, diapolycopene oxygenase; CrtNc, 4,4’-diapolycopenoate synthase; CrtQ, 4,4’-diaponeurosporenoate glycosyltransferase; CrtO, Glycosyl-4,4’-diaponeurosporenoate acyltransferase; CrtB, phytoene synthase; CrtI, phytoene desaturase; CrtY, lycopene beta-cyclase; CrtYB, bifunctional lycopene cyclase/phytoene synthase; CrtW, beta-carotene ketolase; CrtZ, beta-carotene hydroxylase; and CrtS, astaxanthin synthase.

All studied *Methylomonas* genomes possessed the entire set of genes encoding enzymes of methyl-D-erythritol phosphate (MEP) pathway for the biosynthesis of precursor molecules, isopentenyl diphosphate (IPP), and dimethylallyl diphosphate (DMAPP) (Figure 4).

No genes were identified for the alternative mevalonate pathway, which is common in archaea, fungi, and animals, and can also be found in some bacteria [11]. All genomes harbored the gene for farnesyl diphosphate (FPP) synthase that catalyzes the sequential addition of the hydrocarbon moieties of dimethylallyl diphosphate (C5) and geranyl diphosphate (C10) to IPP (C5) to give FPP (C15) [63]. The *crtM* gene, encoding a homologue of the diapophytoene synthase in *Staphylococcus*, was not found in any of the genomes. In contrast, a single-copy gene for squalene/phytoene synthase family protein, containing SQS_PSY domain (PF00494, Pfam database), was identified in a cluster with the gene encoding squalene-hopene cyclase in all examined *Methylomonas* genomes. SQS catalyzes a two-step conversion of two FPP molecules into squalene. The *hpnCDE* genes, which are also responsible for the synthesis of squalene, were not detected in *Methylomonas* genomes. Strain MW1 had no genes for the synthesis of C30 carotenoids. In all pigmented strains, genes involved in the subsequent steps of carotenoid biosynthesis were identified as a clustered unit, which included *crtN*, *crtNb*, and *crtNc.* Consequently, CrtN serves as the first amino oxidase enzyme in the pathway, catalyzing the first desaturation of the C30 backbone with the formation of 4,4’-diapolycopene. Diapolycopene oxygenase (CrtNb) catalyzes the oxidation of 4,4’-diapolycopene to yield 4,4’-diapolycopene-4,4’-dial. Then, the oxidative activity of 4,4’-diapolycopenoate synthase (CtrNc) leads to the formation of 4,4’-diapolycopene-4,4’-dioate. As expected, the non-pigmented strain MW1^T^ had no genes for C30 carotenoid biosynthesis. To examine the pattern of carotenoid-encoding genes in other *Methylomonas* representatives, we included all available *Methylomonas* genomes from Genbank’s Refseq Database in the analysis. The metabolic route leading to the production of 4,4’-diapolycopene-4,4’-dioate, encoded by *crtN*, *crtP*, and *crtNc* genes, was identified in all analyzed genomes except that of strain MW1^T^. We also examined the genomes for the presence of genes related to C40 carotenoid pathways. The gene encoding the squalene/phytoene synthase family protein is the sole identified candidate for the synthesis of the C40 molecule. Genomes of strains MP1 and MO1 also harbor genes encoding phytoene desaturases, which are responsible for the conversion of colorless phytoene into lycopene.

## 4. Discussion

In this study, we identified carotenoids produced by five strains of *Methylomonas* species which displayed a spectrum of pigmentation ranging from white and yellow to orange and red. We also sequenced genomes and identified genome-encoded metabolic routes of carotenoid biosynthesis. The biosynthesis of C30 carotenoids in prokaryotes was originally elucidated on a *Staphylococcus aureus* model where it starts with the enzyme CrtM, 4,4’-diapophytoene synthase, which catalyzes the head-to-head condensation of two molecules of farnesyl diphosphate (FPP) into the C30 carotenoid 4,4’-diapophytoene [64,65]. However, CrtM is specific to *Firmicutes*, meaning that an alternative biochemical route should exist in other carotenoid-producing bacteria. Recently, squalene was proposed as a precursor for biosynthetic pathway of C30 carotenoids [66]. Squalene is an intermediate in the biosynthesis of sterols, hopanoids, and related pentacyclic triterpenes in bacteria [67]. The presence of the squalene/phytoene synthase-encoding gene in all *Methylomonas* genomes may suggest that they also utilize the C30 backbone of squalene for carotenoid biosynthesis. Previously, squalene synthases from yeast, humans, and bacteria subjected to directed evolution were shown to produce the C30 carotenoid backbone, dehydrosqualene [68]. Dehydrosqualene, or 4,4’-diapophytoene, is converted to 4,4’-diapolycopene through the enzymatic activity of 4,4’-diapophytoene desaturase. Notably, among publicly available *Methylomonas* genomes, strain MW1^T^ stands out as the sole example lacking genes for carotenoid biosynthesis. This distinct feature suggests that strain MW1^T^ may represent the only non-pigmented phenotype among currently known *Methylomonas* bacteria. The closest phylogenetic relative to strain MW1^T^, *M. albis*, was characterized as pink-pigmented methanotroph [69]. All other studied strains harbor genes encoding CrtN, CrtNb, and CrtNc necessary for the biosynthesis of 4,4’-diapolycopene, 4,4’-diapolycopene-4,4’-dial, and 4,4’-diapolycopene-4,4’-dioate [33]. CrtN is involved in the synthesis of the yellow carotenoid 4,4’-diaponeurosporene or the red carotenoid 4,4’-diapolycopene. Notably, the introduction of *crtM* and *crtN* genes from *S. aureus* into *B. subtilis* led to the accumulation of two C30 carotenoids, 4,4’-diapolycopene and 4,4’-diaponeurosporene, which resulted in the yellow pigmentation of the engineered strain [70]. From a genomic point of view, the accumulation of these compounds in different proportions may explain the yellow, orange, and red color of studied *Methylomonas* phenotypes.

According to the HPLC analysis of pigment extracts, carotenoids were produced by all strains except for strain MW1^T^. This strongly correlates with the absence of carotenoid pathway genes in the complete genome of strain MW1 and is consistent with observations that the culture of strain MW1 remained colorless regardless of cultivation conditions. C30 carotenoids were predominant in the pigment extracts of all other studied *Methylomonas* strains, which was expected from the genome analysis due to the presence of genome-encoded metabolic pathway leading to the production of 4,4’-diapolycopene-4,4’-dioic acid (C30). In a previous study, another *Methylomonas* bacterium, strain 16a (ATCC PTA-2402), produced esters of both 4,4-diapolycopene-4-oic acid and 4,4-diapolycopene-4,4-dioic acid [33]. The nonsaponified carotenoids from this strain migrated slightly differently compared to the 4,4’-diapolycopene-4,4’-dioic acid and 4,4’-diapolycopene-4-oic acid found in *Methylobacterium rhodinum* by using HPLC analysis. C35 carotenoids were exclusively present in pigment fractions of strains MV1 and MP1^T^. Previously, the co-expression of *S. aureus* CrtM and *Erwinia* geranylgeranyldiphosphate (GGDP) synthase in *Escherichia coli* led to the production of carotenoids with the asymmetrical C35 backbone as the result of the uptake of GGDP by CrtM when FDP was depleted [40]. Considering enzyme promiscuity, the C35 carotenoids in our study could have been synthesized from GGDP and FDP by SQS, followed by further desaturation by *crtN*. Remarkably, C40 carotenoids were detected in pigment extracts from strains MP1^T^, MY1, and MV1. Previously, another *Methylomonas* bacterium, strain ZR1, was shown to synthesize lycopene [25]. As far as we know, the genetic determinants of C40 carotenoid pathways have never been reported for *Methylomonas* bacteria. In this study, genome analysis did not definitively confirm whether *Methylomonas* strains can produce the C40 carotenoid phytoene. One of the possible explanations is that all studied genomes harbor the squalene/phytoene synthase family protein with the SQS_PSY domain (PF00494, Pfam database). Squalene synthase and phytoene synthase share a number of functional similarities [71,72]. It is tempting to speculate that squalene synthase from *Methylomonas* may also accept GGPP molecules as a substrate to produce phytoene. Notably, some *Methylomonas* strains, in addition to having 4,4’-diapophytoene desaturases, also possess phytoene desaturases, enabling them to synthesize the C40 carotenoid lycopene. The growing data on the production of various C35 and C40 carotenoid compounds by *Methylomonas* species offer avenues for investigating the diversity of carotenoid synthesis pathways in these bacteria.

The capability for fast growth on methane is a necessary requirement in certain biotechnological applications, such as single-cell protein production. All five studied strains were capable of robust growth on methane. However, the highest growth rates (0.25–0.33 h^−1^) were observed for strains MV1, MY1, and MP1^T^, which are comparable to those of thermotolerant *Methylococcus* bacteria [24]. Notably, the growth rates of some *Methylomonas* methanotrophs were reported to be even higher than those of *Methylococcus* strains [24,25]. Pigmented methanotrophs of the genus *Methylomonas* offer an alternative to commonly used non-pigmented *Methylococcus capulatus* for the production of single-cell protein enriched with carotenoids. A number of carotenoids are recognized for their ability to influence the antioxidative state and immune system, which leads to improved disease resistance, growth performance, survival, and egg quality in farmed fish without causing any cytotoxicity or side effects [73]. Many fish species accumulate carotenoids in their integuments and muscles, contributing quality criteria to meet consumer demands of aquaculture products [74]. Thus, the biomass of carotenoid-producing *Methylomonas* bacteria may represent a promising source of fish feed in modern aquaculture.

## 5. Conclusions

Overall, this study provided insights into the carotenoid biosynthesis in *Methylomonas* bacteria. The major carotenoids identified in the pigmented strains were 4,4’-diaplycopene-4,4’-dioic acid (C30) and 1,1’-dihydroxy-3,4-didehydrolycopene (C40). Genome analysis revealed the presence of C30 carotenoid biosynthesis pathways leading to 4,4’-diaplycopene-4,4’-dioic acid. Notably, strain MW1^T^ lacked carotenoid biosynthesis genes and may represent the only colorless phenotype among *Methylomonas* bacteria. Several strains also possess phytoene desaturases, which explains the presence of lycopene compounds in the pigment extracts. Thus, *Methylomonas* bacteria possess the ability to naturally synthesize both C30 and C40 carotenoids. Novel isolates display robust growth on methane and represent promising candidates for certain industrial applications, such as single-cell protein production enriched with carotenoids. Further research into the metabolic pathways and genetic manipulation of *Methylomonas* bacteria may unlock their full potential in biotechnology.

## Figures and Tables

**Table 1:** Isolation sources and some characteristics of *Methylomonas* strains used in this study.

Strain	GenBank 16S rRNA	Sampling Site	Closest Relative (% Similarity to 16S rRNA)	Color	Cell Size, Wide/Long, μm	Growth Temperature Range (Optimum), °C	Growth Rate in Batch Culture, h^−1^
MP1	ON819564	Unnamed lake, Krasnodar region, Russia (N 44.42°; E 39.18°)	*M. rapida* (100)	pink to red	1.10 ± 0.03/2.10 ± 0.08	8–45 (35)	0.33
MY1	OR234855		*M. koyamae* Fw12E-Y (100)	yellow	1.5 ± 0.07/1.9 ± 0.1	8–37 (30)	0.29
MO1	OR234854	Meshchersky pond, Moscow, Russia (N 55.67°; E 37.40°)	*M. denitrificans* FJG1 (99.65)	orange	0.9 ± 0.04/1.3 ± 0.07	5–37 (32)	0.22
MV1	OR234856	Unnamed pond, Krasnodar region, Russia (N 44.42°; E 39.18°)	*M. koyamae* Fw12E-Y (97.56)	pink	0.7 ± 0.03/2.2 ± 0.09	4–38 (30)	0.25
MW1	OR237191	Khosta river, Krasnodar region, Russia (N 43.53°; E 39.97°)	*M. methanica* S1 (97.29)	white	0.9 ± 0.04/1.5 ± 0.2	10–35 (30)	0.13

**Table 2:** Carotenoid composition in studied strains of *Methylomonas* spp., mol %.

Retention Time, min	Peak Absorption Maxima, nm	Identity	Formula	Strains				
				MY1	MO1	MP1	MW1	MV1
5.7	467/489/519	1,2-Dihydro-3,4-dehydrolycopene	C_40_H_56_	–	–	–	–	5.0
6.5	469/498/527	4’-Apo-3,4-didehydrolycopene	C_35_H_46_	–	–	2.9	–	4.8
7.0	465/489/521	4,4’-Diaplycopene-4,4’-dioic acid	C_30_H_36_O_4_	68.7	78.3	67.9	–	90.2
7.3	456/484/513	1,1’-Dihydroxy-3,4-didehydrolycopene	C_40_H_58_O_2_	31.3	21.7	18.0	–	–
7.8	470/508/540	Tetradehydrolycopene	C_40_H_52_	–	–	1.8	–	–
9.0	451/484/510	4,4’-Diaplycopenoic acid	C_30_H_38_O_2_	–	–	9.3	–	–

**Table 3:** Sequencing statistics.

	Characteristics	Strain MO1	Strain MV1	Strain MY1	*“M. montana”* MW1^T^	*M. rapida* MP1^T^
Illumina	Number of reads	2,022,040	3,782,980	3,724,476	3,643,858	1,304,732
	Total bases, Gb	0.6	1.1	1.1	1.1	0.4
	Mean read length, bp	301	301	301	301	301
Nanopore	Number of reads	258,525	216,073	211,318	266,732	150,333
	Total bases, Gb	1.2	1.4	1.4	1.6	0.9
	Mean read length, bp	4497.9	6464.9	6411	6126.4	6263.5
Total coverage		352	468	500	589	290

**Table 4:** General genome characteristics of studied *Methylomonas* strains.

	Strain MO1	Strain MV1	Strain MY1	“*M. montana*” MW1^T^	*M. rapida* MP1^T^
Genome size (Mb)	5.03	5.42	4.95	4.63	4.60
Contigs	2	10	1	1	1
G + C content (mol %)	51	55.5	56	52	52.5
CDS	4556	4820	4469	4296	4343
Repeat region	0	2	5	2	3
tRNA	46	49	52	47	51
5S, 16S, 23S	3, 3, 3	3, 3, 3	3, 3, 3	3, 3, 3	4, 4, 4
pMMO operon	1	1	1	1	1

## Data Availability

The datasets used and/or analyzed during the current study are available from the corresponding author on reasonable request.
